# Soil and forest structure predicts large-scale patterns of occurrence and local abundance of a widespread Amazonian frog

**DOI:** 10.7717/peerj.5424

**Published:** 2018-08-09

**Authors:** Anthony S. Ferreira, Robert Jehle, Adam J. Stow, Albertina P. Lima

**Affiliations:** 1 Programa de Pós-Graduação em Ecologia, Instituto Nacional de Pesquisas da Amazônia, Manaus, Amazonas, Brazil; 2 School of Environment and Life Sciences, University of Salford, Salford, UK; 3 Department of Biological Sciences, Macquarie University, Sydney, NSW, Australia; 4 Coordenação de Biodiversidade, Instituto Nacional de Pesquisas da Amazônia, Manaus, Amazonas, Brazil

**Keywords:** Amazonia, Ecology, *Allobates femoralis*, Environmental heterogeneity, Ecological limiting factors, Tropical forest

## Abstract

The distribution of biodiversity within the Amazon basin is often structured by sharp environmental boundaries, such as large rivers. The Amazon region is also characterized by subtle environmental clines, but how they might affect the distributions and abundance of organisms has so far received less attention. Here, we test whether soil and forest characteristics are associated with the occurrence and relative abundance of the forest-floor dwelling Aromobatid frog, *Allobates femoralis*. We applied a structured sampling regime along an 880 km long transect through forest of different density. High detection probabilities were estimated for *A. femoralis* in each of the sampling modules. Using generalized linear mixed-effects models and simple linear regressions that take detectability into account, we show that *A. femoralis* is more abundant in open forests than in dense forests. The presence and relative abundance of *A. femoralis* is also positively associated with clay-rich soils, which are poorly drained and therefore likely support the standing water bodies required for reproduction. Taken together, we demonstrate that relatively easy-to-measure environmental features can explain the distribution and abundance of a widespread species at different spatial scales. Such proxies are of clear value to ecologists and conservation managers working in large inaccessible areas such as the Amazon basin.

## Introduction

The distribution of species is often fragmented, with favorable habitats being separated from each other by unsuitable habitats (Krebs, 1972; Hanski, 1999). Nevertheless, determining how habitat heterogeneity influences the distribution and abundance of species at various geographic scales remains one of the major challenges in ecology and conservation biology (Leibold et al., 2004; Fraterrigo, Wagner & Warren, 2004; Tews et al., 2004; McGarigal et al., 2016). Species interact with each other at fine scales, and habitat heterogeneity within a landscape moderates the broad-scale consistency of such interactions, producing variation in environmental effects at intermediate scales (Lawton, 1999). A persistent problem is explaining how abiotic and biotic factors affect the distributions of species across spatial scales which are hierarchical in nature (Fraterrigo, Wagner & Warren, 2004). Recent work has disentangled historical and environmental determinants for the spatial turnover of species assemblage compositions (Ricklefs & Schluter, 1993; Holyoak, Leibold & Holt, 2005; Bitar et al., 2017). However, for single species, the main focus has been on predicting entire ranges by extrapolating from local data on habitat requirements (e.g., using species distribution models, see Elith & Leathwick, 2009; Zurell et al., 2016), for which an understanding of the potential hierarchy of controls on species’ niches is critically important (Araujo & Luoto, 2007; Fraterrigo, Wagner & Warren, 2004). Studies that use empirical data to quantify habitat associations for single species across biogeographic scales, however, are surprisingly rare.

In the Amazon basin, biogeographic and large-scale ecological studies are particularly challenging, due to difficulties of access and a general lack of baseline knowledge (Tuomisto et al., 2003; Laurance et al., 2004; Betts, Malhi & Roberts, 2008; Gardner et al., 2008; De Fraga et al., 2014). To explain patterns of endemism, marked ecological barriers such as large rivers have been found to predict the distribution of many taxa (Cracraft, 1985; Aleixo, 2006; Araripe et al., 2008; Ribas et al., 2012; Dias-Terceiro et al., 2015; Nazareno, Dick & Lohmann, 2017; Oliveira, Vasconcelos & Santos, 2017). While sharp environmental boundaries clearly influence the abundance and occurrence of organisms, the effect of more gradual clines of biotic and abiotic features are less appreciated (Quesada et al., 2012; Cintra et al., 2013; Emilio et al., 2013; Schietti et al., 2016). Under such a scenario, the abundance of organisms and patterns of local adaptation are shaped by continuous environmental changes across the landscape (Endler, 1977; Leite & Rogers, 2013; Dias-Terceiro et al., 2015; Bitar et al., 2017).

Anurans are useful models to evaluate biogeographic and ecological determinants of species assemblages in tropical ecosystems owing to their high diversity, low vagility and specific environmental requirements (Zimmerman & Bierregaard, 1986; Ernst & Rödel, 2008; Menin et al., 2007; Keller et al., 2009). Furthermore, based on their life histories, groups of species can be assigned to specific guilds (e.g., lotic and lentic aquatic breeders, forest-floor dwellers and canopy species; for case studies from Amazonia see Zimmerman & Simberloff, 1996; Menin et al., 2007; Rojas-Ahumada, Landeiro & Menin, 2012; Landeiro, Waldez & Menin, 2014; Dias-Terceiro et al., 2015; Bitar et al., 2017). Related species may share behavioral, physiological, and morphological traits because of common ancestry, rather than as a result of being exposed to similar selection pressures and convergent evolution (Huey, 1987; Losos, 1990; Cadle & Greene, 1993; Zimmerman & Simberloff, 1996). Given that some habitat requirements are therefore likely to be shared by all individual species of a guild, it is remarkable that relatively little is known about habitat associations of particular species across significant parts of their range (but see e.g., Jorge et al., 2016).

Many anurans associate with standing water, so their populations are often patchily distributed across the landscape (Smith & Green, 2005). Therefore, a population size estimate is often unattainable as a metric for monitoring population status across large scales, due to its variability and work involved in collecting the data at each patch (Smith et al., 2014). Documenting patch (site) occupancy is a more practical option because it can be measured using presence/absence surveys, utilizing each site as a sampling unit (MacKenzie et al., 2002). Moreover, the identification of accessible and stable environmental features that reflect specific habitat requirements would circumvent the problem of directly measuring standing water bodies, which are often ephemeral, and therefore difficult to record.

The present study identifies and characterizes important environmental parameters linked to the distribution and relative abundance of a widespread Amazonian forest-floor anuran, the Aromobatid frog *Allobates femoralis*, using a structured sampling regime spanning an 880 km environmental gradient across an interfluvial landscape. We focus on structural forest features and soil characteristics as surrogates for the species’ microhabitat requirements, and show that these features are able to predict both its regional occurrence as well as its large-scale relative abundance. Our findings suggest that the distribution and relative abundance of *A. femoralis* is shaped by gradual ecological clines.

## Material and Methods

### Study species

The brilliant-thighed poison frog *A. femoralis* (Boulenger, 1883; Anura: Aromobatidae Grant et al., 2017; ♂ snout-vent length = 28–33 mm; ♀ snout-vent length = 33–35 mm) is widely distributed in non-flooded primary forests of the Amazon Basin and Guiana Shield in Brazil, Bolivia, Peru, Ecuador, Colombia, Guyana, Suriname, French Guiana and Venezuela (Lescure & Marty, 2000; Lima et al., 2006; Amézquita et al., 2009; Barrio-Amorós & Santos, 2010), although phylogeographic and taxonomic studies suggest that it comprises a suite of cryptic species (Grant et al., 2006, 2017; Fouquet et al., 2007; Santos et al., 2009; Simões, Lima & Farias, 2010). *A. femoralis* is active in leaf litter or on fallen tree trunks on the forest floor, with males exhibiting territorial behavior (Roithmair, 1992; Montanarin, Kaefer & Lima, 2011). Females lay eggs on dead leaves in male territories during the rainy season, and males use water bodies ranging from shed palm bracts, Brazil-nut capsules, puddles on fallen tree trunks, peccary wallows and temporary puddles on the forest floor to deposit tadpoles after hatching (Roithmair, 1994; Gascon, 1995; Ringler, Ursprung & Hödl, 2009; Beck, Thebpanya & Filiaggi, 2010; Ringler, Hödl & Ringler, 2015; Pašukonis et al., 2016, 2017). The availability and location of sites for tadpole deposition influences year-to-year displacement of individuals that survive more than one breeding season (Ringler, Ursprung & Hödl, 2009). The ephemeral occurrence of suitable bodies of water also sometimes forces male *A. femoralis* to deposit tadpoles more than 180 m away from their territories (Ringler et al., 2013), to which they reliably return (Pašukonis et al., 2013, 2014). Over the last two decades, *A. femoralis* has been used as a model species to address questions on diversification (e.g., Lougheed et al., 1999; Simões et al., 2008; Amézquita et al., 2009), sexual selection and parental care (Ringler et al., 2015, 2016, 2017a; Ursprung et al., 2011; Pašukonis et al., 2016, 2017), movement ecology and spatial cognition (Pašukonis et al., 2016; Beck et al., 2017), and communication (Hödl, Amézquita & Narins, 2004; Amézquita, Castellanos & Hödl, 2005; Amézquita et al., 2006; Narins et al., 2005; Betancourth-Cundar et al., 2016; Ringler et al., 2017b).

### Study area

**Figure 1:**
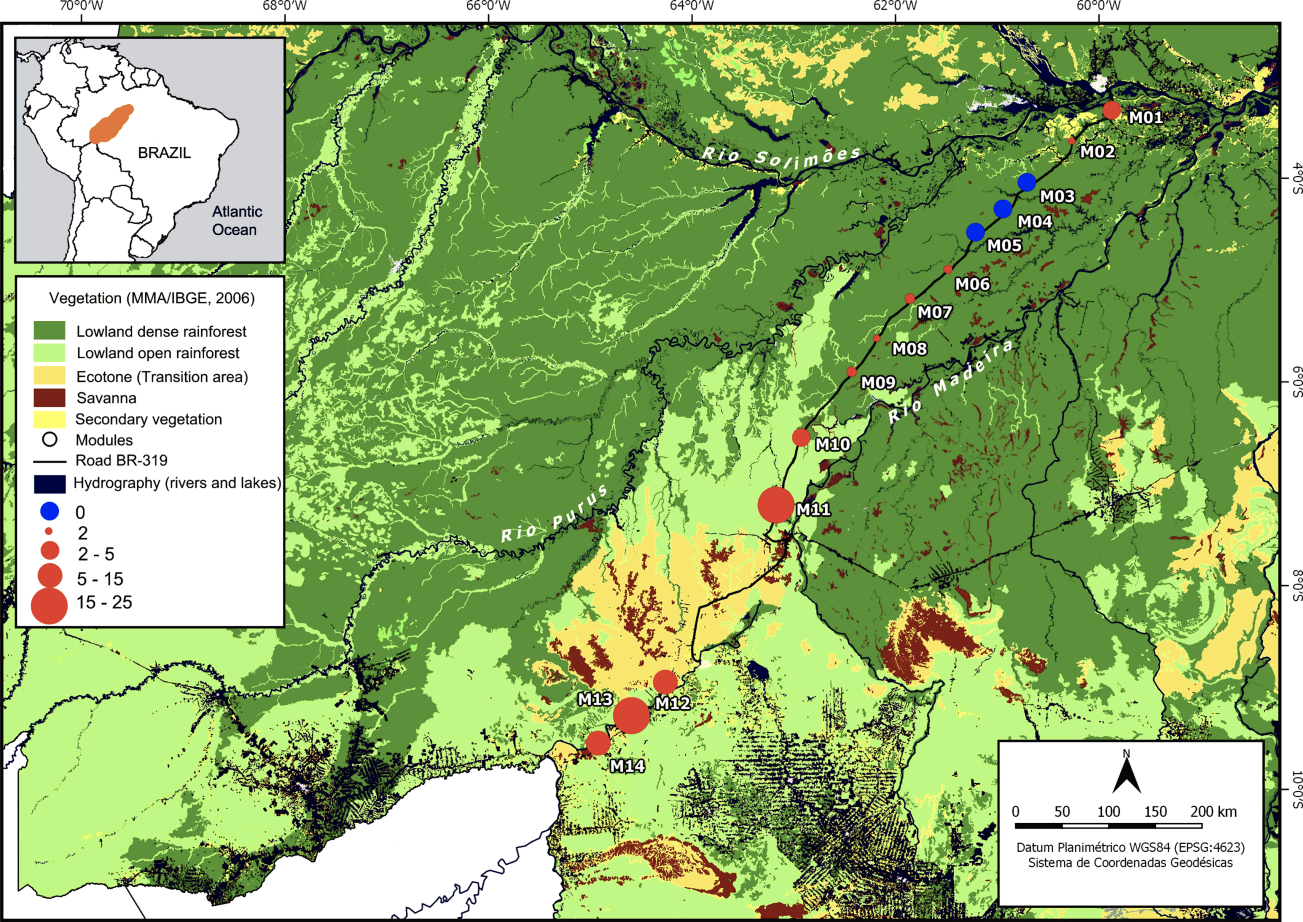
Purus-Madeira interfluve showing modules locations with the two main types of phytophysiognomy and the range of means of the relative abundance of *A. femoralis*. Purus-Madeira interfluve showing module locations (M1–M14), and the two main types of forest phytophysiognomy: lowland dense rainforest to the northeast (dark green) and lowland open rainforest to the southwest (light green). Red circles indicate the presence of *A. femoralis* with sizes representing relative abundances; blue circles indicate absence of *A. femoralis*. The scale of the symbols represents the range of means of the relative abundance of *A. femoralis* in the modules when present. The modules are approximately 50–60 km apart, with the exception of the distance between M11 and M12 which are separated by open transition forest and savanna. Map created in QGis 2.14 Essen (http://qgis.osgeo.org).

The Purus-Madeira interfluve (PMI) is located on the eastern boundary of the Inambari area of endemism in central-southern Amazonia, delimited by the Amazon, Purus and Madeira rivers and covering about 15.4 million hectares (Fearnside et al., 2009; Fig. 1). It is of sedimentary origin in its northeastern parts (Late Pleistocene-Early Holocene see Sombroek, 2000; Rossetti, Toledo & Góes, 2005), where the water table is closer to the surface and large areas are waterlogged by temporary small streams during the rainy season (Fan & Miguez-Macho, 2010; Schietti et al., 2016). Soils are mainly plinthosols characterized by poor drainage; the predominant texture is silt and fine sand in the northeast (Cintra et al., 2013; Martins et al., 2014) and podzolic soils with a predominant texture of clay and sand in the southwest (IBGE, 1997). The vegetation is classified as humid tropical lowland rainforest, composed of lowland dense rainforest in the northeast, with an about 40 m high canopy and frequent occurrence of palms in the understory, and lowland open rainforest with an about 40 m high canopy in the southeast (IBGE, 1997; Sousa, 2007). Considerable areas of savanna and transition between lowland open forest-savanna are present in the extreme southwest (Fig. 1). In the northeast of the PMI, forests are characterized by a lower basal area, lower canopy heights and lower mean wood density (lowland dense forests) compared to the central and southeast sites (lowland open forests), associated with an increase in rainfall seasonality and a change in soil structure (Sombroek, 2000; Cintra et al., 2013; Schietti et al., 2016). Rainfall is seasonal and heaviest between November and May. The mean annual precipitation ranges from 2,100 mm in the southwest to 2,800 mm in the northeast (Cintra et al., 2013; Alvares et al., 2014). Elevation ranges from 27 to 80 m above sea level (Sombroek, 2000). Temporary ponds occur in lower areas during the rainy season and are formed by undulating terrain (Rossetti, Toledo & Góes, 2005; Ferrão et al., 2018). A more detailed description of the study area is presented by Cintra et al. (2013) and Schietti et al. (2016).

### Sampling design

The BR-319 Highway has not been maintained as a thoroughfare since 1998, but still allows access to a large section of the PMI. The implementation of standardized sampling sites along this highway through the RAPELD (Rapid Assessment for Long Duration Ecological Projects; Magnusson et al., 2013) system has generated a large amount of environmental and biotic information (Levis et al., 2012; Baccaro et al., 2013; Emilio et al., 2013; Cintra et al., 2013; Martins et al., 2014; Schietti et al., 2016; Ferrão et al., 2016; 2018; De Abreu, Schietti & Anciães, 2018). For the present study, we collected data in 152 plots at 14 RAPELD research sites (modules, M) which are spread along a 880-km-long transect (Fig. 1; Table S1, see Magnusson et al., 2013 for more details). Standard RAPELD modules consist of two straight parallel five km long trails starting at one km distance from each other, with five plots of 250 × 60 m (∼1.5 ha) that follow altitudinal isoclines to minimize within-plot environmental heterogeneity installed on each trail, at distances of one km (Fig. S1). A total of 11 modules were installed along the BR-319 Highway (M01–M11), with plots established at least one km from the road to avoid secondary forests. Three other modules are near the left bank of the Madeira River (M12–M14), with seven 250 m plots resulting in a total of 14 plots per module and installed in the same way as those along the BR-319 Highway. Detailed descriptions of RAPELD sampling units throughout the Amazon basin are available at https://ppbio.inpa.gov.br.

### Allobates femoralis sampling

We used time- and space-limited visual sampling (adapted from Crump & Scott, 1994; 2 min searches every 10 m along the 250 m long plots) and auditory searches (using playback to stimulate male responses) to quantify the relative abundance of *A. femoralis*. Each sampling session lasted about one hour along the 250 m long plot central line, and was undertaken by two experienced observers. The presence or absence of *A. femoralis* was recorded in segments of 10 m. As only a single record per segment was made, the maximum number of records was 25 per plot. We were careful not to record the same individual more than once on the same segment or in neighboring segments. Sampling was carried out during the breeding season, which coincides with the regional rainy season (Kaefer et al., 2012; Ferrão et al., 2018), between December and February 2010–2015.

Data were collected during the daily periods of peak vocalization for the species (7:00–10:00 a.m. and 14:00–18:00 p.m., Kaefer et al., 2012). To determine if *A. femoralis* was present, we recorded data on calling males following the audio strip transect method outlined by Zimmerman (1994). The *A. femoralis* advertisement call is one of the best studied anuran vocalizations (Narins, Hödl & Grabul, 2003; Hödl, Amézquita & Narins, 2004; Amézquita, Castellanos & Hödl, 2005; Amézquita et al., 2006, 2009; Göd, Franz & Hödl, 2007; Simões et al., 2008; Ringler et al., 2017b), and in the study area is a trill composed of four whistle-like notes with ascending frequency modulation. Calling males of *A. femoralis* could be heard from a distance of 30 m, and are easily recognized. Each of the 152 plots was sampled once per field season, giving a total of four surveys per site in total. To avoid temporal bias, two sampling sessions followed the directions M1–M14 and M14–M1, respectively, with the remaining two sampling sessions not undertaken in consecutive order. The research project was approved by the appropriate governmental bodies: Ministério do Meio ambiente (MMA), the Instituto Chico Mendes de Conservação da Biodiversidade (ICMBio license 13777) and the Sistema de Autorização e Informação em Biodiversidade (SISBIO license 7836-1) for the sampling of *A. femoralis*. All sampling procedures were approved by the ethics animal welfare committees of the Instituto Nacional de Pesquisas da Amazônia (CEUA/INPA: 041/2015) in accordance with established scientific practice guidelines and current Brazilian legislation.

### Environmental variables

To represent environmental effects on the distribution and relative abundance of *A. femoralis* along the PMI, we used physical soil parameters (sand, clay and silt contents) and forest structure (basal area and number of trees, see Table S2). In all plots, soil samples were extracted with an auger every 50 m along the central 250 m long transect to a depth of 10 cm (a total of six samples per plot). Samples were kept in sealed plastic bags for 2–5 days, air dried at ambient temperature, and mixed to form one composite sample per plot (Cintra et al., 2013). Soil physical structure was analyzed following a standard protocol of total dispersion, using sodium pyrophosphate to obtain relative clay, sand and silt contents (Donagema et al., 2011). Percentage of sand was determined with a 0.053 mm mesh sieve (tensile bolting cloth 16), dividing the remaining fraction into silt and clay (Donagema et al., 2011). The proportion of clay was determined by separating particles of 20 μm from other particles, and the proportion of silt was determined by the difference between clay and sand values; for a full description of the methodology see Quesada et al. (2010) and Donagema et al. (2011).

Forest structure was represented by the basal area and the hierarchical sum of the number of trees and palms in three size classes: (1) in a band (left side of the center line) of 250 × 1 m (0.025 ha), counting all stems with diameter at breast height (DBH) ≥ 1 cm; (2) in a band of 250 × 20 m (0.5 ha), counting all stems with DBH ≥ 10 cm; and (3) in a band of 250 × 40 m (one ha), counting all stems with DBH ≥ 30 cm (Magnusson et al., 2005). Tree diameters were measured with a diametric tape to mm precision (Schietti et al., 2016). Total plot basal area was calculated by the sum of the transverse areas of all trees as π(DBH)^2^/4. Data for modules M1–11 were obtained from Schietti et al. (2016), whereas data for modules M12–14 were previously unpublished. Forest structure has previously been identified as an important factor affecting the distribution and abundance of frogs in Amazonia (Menin et al., 2007; Menin, Waldez & Lima, 2011; Landeiro, Waldez & Menin, 2014; Ferrão et al., 2018).

### Data analysis

To estimate occupancy and detection probabilities of *A. femoralis* for each module, we used a multi-season occupancy model based on four seasons of sampling and presence-absence data without covariates in the program PRESENCE v.12.10 (MacKenzie et al., 2003). Models developed for estimating occupancy can account for imperfect detection by using data from repeat surveys to discriminate between a species being either truly absent, or present but undetected (MacKenzie et al., 2003; 2006; Tyre et al., 2003). The probability of occupancy is only estimated for the first season in a multi-season analysis, with occupancy parameters for the subsequent seasons being derived using a recursive equation (MacKenzie et al., 2003). Detection probabilities might vary slightly among areas within each module as a function of change in habitat features. However, because we were interested in estimating detectability at the scale of tens of kilometres for each module, we used a model without covariates. We excluded from the model the three modules where *A. femoralis* has never been found (M3–M5).

Taking detection probabilities per module into account, we used Generalized Linear Mixed-Effects Models (GLMMs) to investigate the variation in relative abundance along gradients with the forest-structure components (basal area and number of trees) and soil texture (sand, clay and silt) as fixed effects. Modules were included in the model as a random effect to account for the nested design (plots within modules, Zuur et al., 2009).

We also used the detectability-corrected data per module to ran simple linear regressions to investigate relationships between the relative abundance of *A. femoralis* with each predictor environmental variable. We used Shapiro–Wilk analyses to test for significant deviation from normality, and Spearman’s coefficient to verify correlations between environmental variables. As M11 is characterized by high variation in silt across plots and a high abundance of *A. femoralis* in three plots appearing as an outlier, separate analyses with and without M11 were undertaken. As the number of records was low in most plots, we used the sum of recorded individuals (instead of the mean) for the four samplings to represent the relative abundance of *A. femoralis* in each plot (following Bueno et al., 2012; Ferrão et al., 2018). To test for spatial autocorrelation among the modules, we used a Moran’s correlogram of geographical distance between pairs of modules and on the residuals of linear regression analysis of dependent variable between pairs of modules.

Statistical analyses were carried out in the statistical platform R 3.2.3 (R Core Team, 2018). GLMM analyses were conducted with the packages lme4 (Bates, Maechler & Bolker, 2015), and the DHARMa package was used for the creation and simulation of scaled (quantile) residuals (Hartig, 2017). The marginal and conditional GLMMs *r*^2^ were calculated using the package MuMIn (Bartoń, 2018) and figures were compiled using the package visreg (Breheny & Burchett, 2017). We used the APE package to test for spatial auto-correlation (Paradis, Claude & Strimmer, 2004). We only show data in figures when the simple linear regressions model was significant at the 0.05 level. Maps were prepared with *QGis 2.14 Essen* (QGIS Development Team, 2016).

## Results

**Figure 2:**
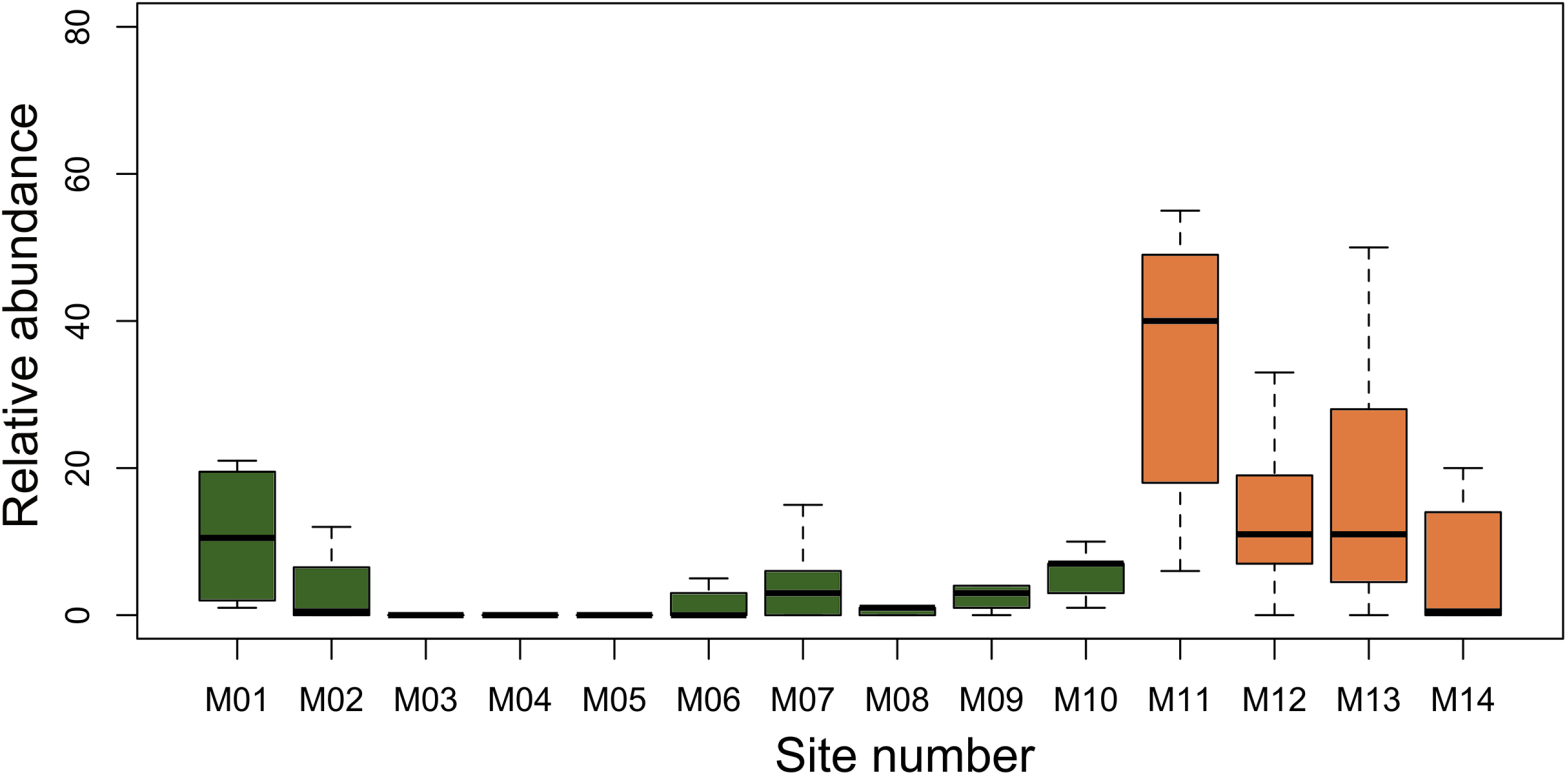
Median of *A. femoralis* relative abundance in the research modules along the Purus-Madeira interfluve. Median, quartiles and maximum and minimum values of *A. femoralis* relative abundance (sum of individuals per plot across all samplings taking detectability into account) in the research modules along the Purus-Madeira interfluve. Green bars (M1–M10) represent modules in the lowland dense rainforest, and orange bars (M11–M14) represent modules in the lowland open rainforest.

We found *A. femoralis* in 11 of 14 modules. The average number of segments per module in which we detected *A. femoralis* varied from 1.5 to 10 in lowland dense rainforest modules when present, while in lowland open rainforest the mean the respective number ranged from 12 to 25 per module (Fig. 1). At the level of plots, the encountered number of *A. femoralis* was on average 80% higher in lowland open rainforest (mean = 15.67; SD = 17.21, Fig. 2) than in lowland dense rainforest (mean = 3; SD = 5.01, Fig. 2).

Estimates from the multi-season model showed that the median proportion of plots predicted to be occupied per module was 0.44 (0.14–0.78), and that median *A. femoralis* detection probabilities were 0.78 (0.48–0.96) across the four surveys (Table 1). There was no correlation between the geographical distance among modules and their dissimilarity in soil structure (sand, clay and silt contents; Moran tests, *p* = 0.12, 0.39, and 0.80, respectively) or forest structure (basal area and number of trees; Moran tests, *p* = 0.70 and 0.40, respectively).

**Table 1:** Occupancy probabilities and detection of *A. femoralis*.

Modules	O.M.	SE_OM_	*p*	SE_*p*_
M1	0.53 (0.00–1.00)	0.42	0.61 (0.00–1.00)	0.32
M2	0.28 (0.00–0.85)	0.29	0.48 (0.06–0.89)	0.21
M6	0.14 (0.00–0.41)	0.14	0.83 (0.52–1.00)	0.15
M7	0.21 (0.00–0.64)	0.20	0.56 (0.24–0.89)	0.17
M8	0.34 (0.01–0.68)	0.17	0.75 (0.51–1.00)	0.12
M9	0.44 (0.06–0.82)	0.19	0.81 (0.31–1.00)	0.25
M1O	0.57 (0.14–1.00)	0.22	0.90 (0.61–1.00)	0.15
M11	0.70 (0.42–0.99)	0.15	0.96 (0.89–1.00)	0.04
M12	0.78 (0.57–1.00)	0.11	0.91 (0.82–0.99)	0.04
M13	0.44 (0.10–0.78)	0.17	0.89 (0.74–1.00)	0.07
M14	0.42 (0.17–0.69)	0.13	0.87 (0.74–1.00)	0.07

**Notes:**

Occupancy probabilities and detection of *A. femoralis* in 11 research sites which consist of two straight parallel 5 km long trails in central-southern Amazonia. O.M., proportion of plots predicted to be occupied in each module with confidence intervals (± 95%); SE, standard error; *p*, probability of detection for *A. femoralis* with confidence intervals (± 95%) for the four samplings in the Purus-Madeira interfluve.

**Figure 3:**
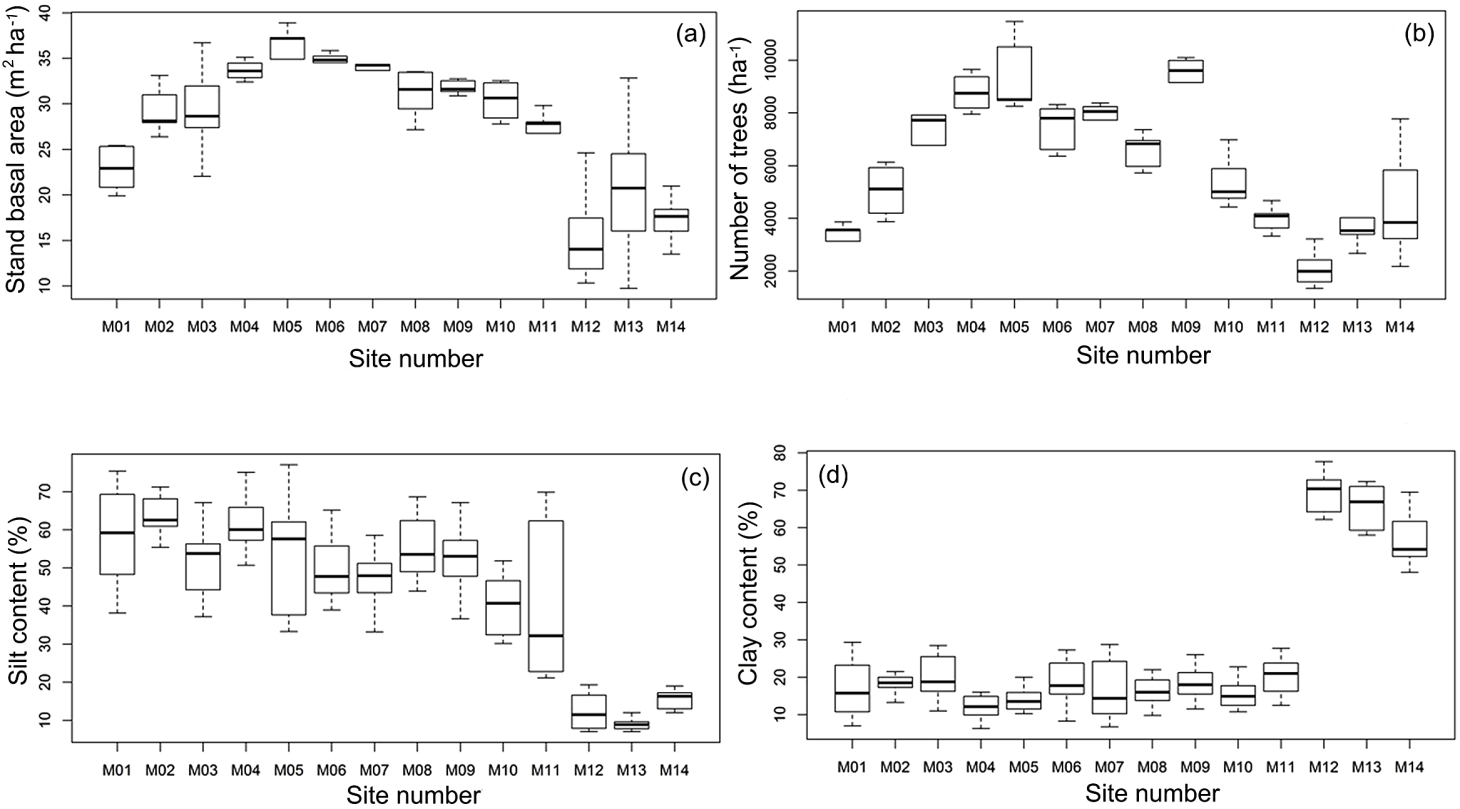
Median, quartiles and maximum and minimum values of environmental variables along the Purus-Madeira Interfluve. Median (black vertical line), quartiles and maximum and minimum values of (A) basal area; (B) number of trees; (C) silt content and (D) clay content across 14 research modules along the Purus-Madeira Interfluve.

The 880 km transect across the PMI was characterized by marked environmental gradients. Tree basal area ranged from 9.73 to 38.90 m^2^ ha^−1^. The number of trees per ha^−1^ varied from 1,335 to 11,475 considering all individuals with dbh ≥ 1 cm (Figs. 3A and 3B), with more trees in the central area of the PMI and fewer trees towards marginal areas in the southwest (Fig. 3B). To the northeast of the PMI, the soil had high levels of silt (average 30–77%), while the soil to the southwest had high clay content (average 50–77%, Figs. 3C and 3D). Module 11 had the highest variation in silt between plots (22–70%, Fig. 3C), and a high relative abundance of *A. femoralis*. Exclusion of this module from the analyses greatly changed the slope, but not the direction, of the curve and masked the relationships with environmental variables. Therefore, we excluded this module from the simple linear regressions (module level), but not from the GLMMs (plot level).

**Figure 4:**
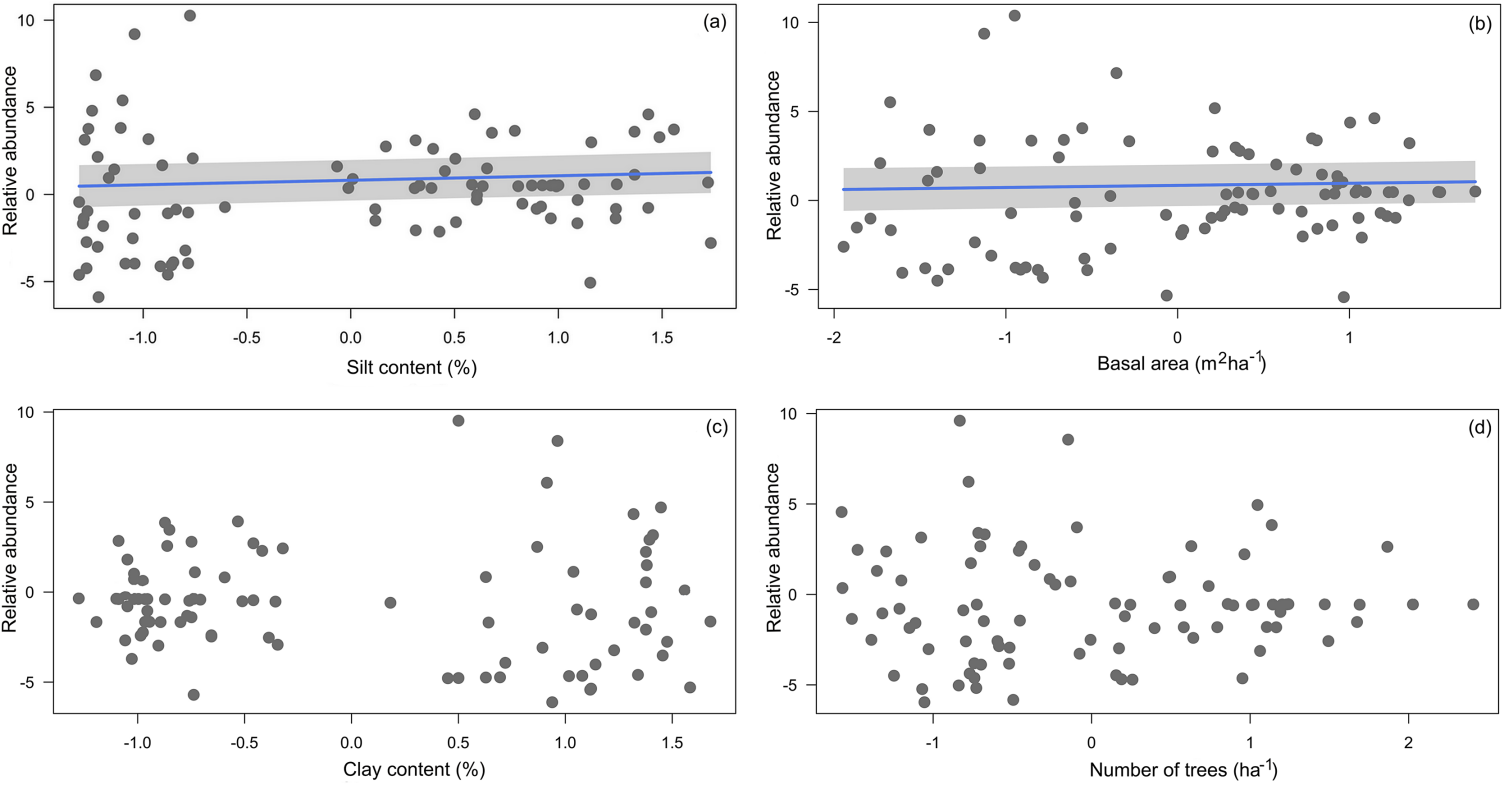
Partial regressions derived from generalized linear mixed-effects models. Partial regressions derived from generalized linear mixed-effects models (GLMMs) investigating the effects of (A) silt content, (B) basal area, (C) clay content and (D) number of trees per plot on *A. femoralis* relative abundance taking detectability into account in forests along the Purus-Madeira interfluve. Fitted lines indicate fixed-effect probabilities < 0.05 in the linear mixed-model analyses.

Four environmental variables were highly correlated (*r* > 0.6) with another variable: clay content with silt content, silt content and clay content with basal area, and basal area with number of trees (Table 2). Soils with more silt coincided with low relative abundance of *A. femoralis* (*p* = 0.006; Fig. 4A), and areas with lower tree basal area had a high relative abundance of *A. femoralis* (*p* = 0.05; Fig. 4B). No interaction was found between clay content and number of trees to determine the relative abundance of *A. femoralis* in the GLMMs (Figs. 4C and 4D). Detailed results of the GLMMs are presented in Table 3.

**Table 2:** Pearson’s correlation coefficients between environmental variables along the Purus-Madeira interfluve.

Variables	Sand	Silt	Clay	Basal area	Number of trees
Sand		−0.30	−0.30	0.38	0.25
Silt	−0.11		−**0.92**	**0.77**	0.54
Clay	−0.39	−**0.82**		−**0.86**	−0.59
Basal area	0.30	**0.64**	−**0.72**		**0.81**
Number of trees	0.37	0.53	−0.54	**0.74**	

**Notes:**

Pearson’s correlation coefficients between environmental variables at the module level (upper right) and plot level (lower left) along the Purus-Madeira interfluve. Bold values correspond to correlated variables (*r* > 0.60, *p* < 0.05).

**Table 3:** Results of generalized linear mixed-effects models—GLMMs.

Dependent variable	Fixed effects	d*f*	AIC	BIC	logLik	*r*^2^_marg_	*r*^2^_cond_	*p*
Relative abundance	Silt + basal area	86	1,138.6	1,148.6	−565.3	0.06	0.47	**0.006 0.05**
	Clay + trees	86	1,147.9	1,157.9	−569.9	0.21	0.44	0.28 0.99
	Sand + basal area	86	1,141.5	1,151.5	−566.7	0.02	0.49	0.09 **0.05**

**Notes:**

Results of generalized linear mixed-effects models (GLMMs) for the relative abundance of *A. femoralis* taking detectability into account as a function of sand, clay and silt contents, basal area and number of trees (fixed effects). Modules was considered a random effect in all model. Marginal *r*^2^ values are for the models adjusted only considering fixed effects and the conditional *r*^2^ corresponds to the full model, including the random effect. The relative contribution of predictors is given by the standardized coefficients of the GLMMs. The probability for each predictor is shown in the sequence as they appear in the models. Standardized coefficients in bold have *p* < 0.05. The outlier module M11 is included in all models.

**Figure 5:**
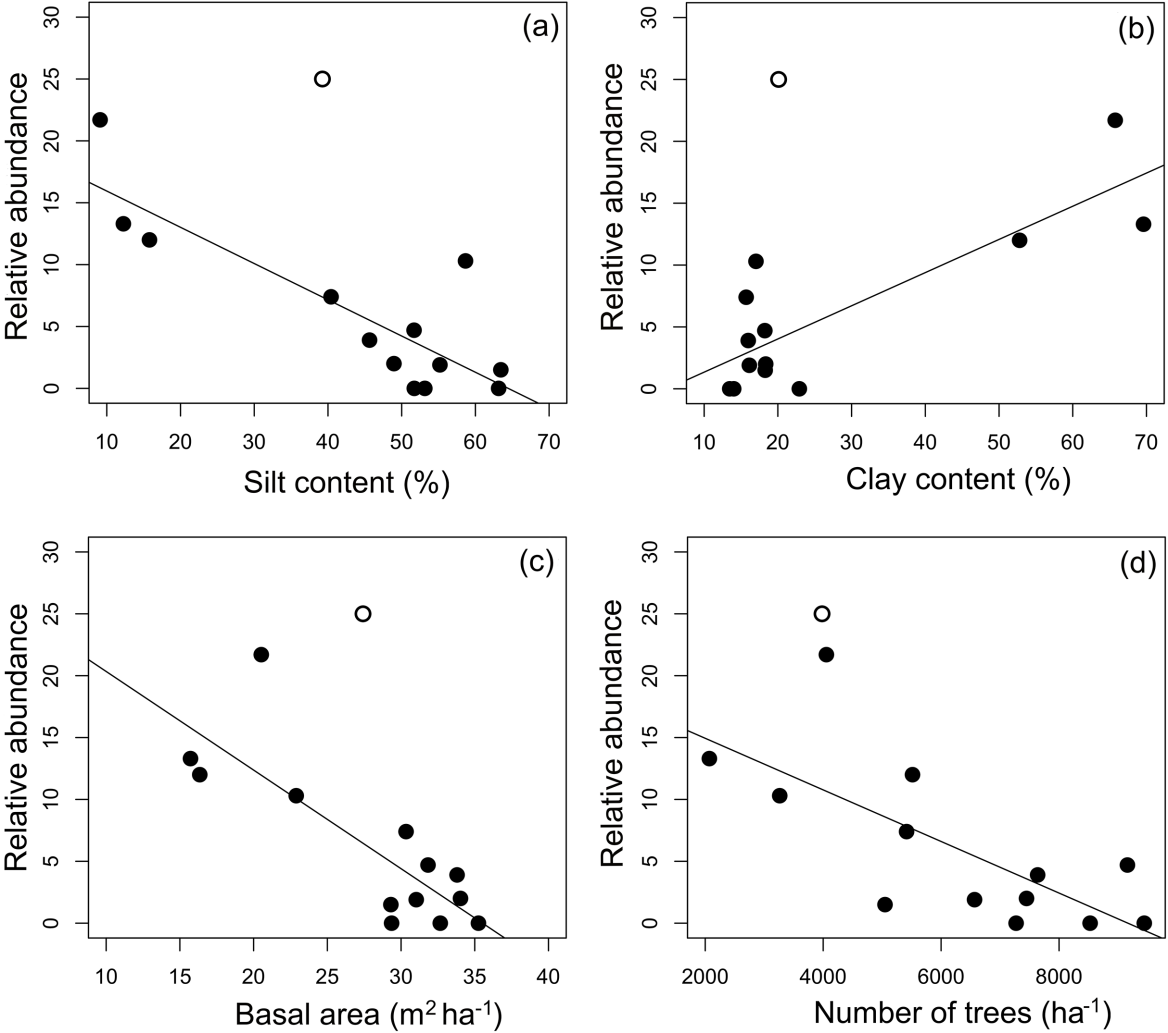
Relationship between mean *A. femoralis* relative abundance and silt and clay contents, basal area and number of trees. Relationship between mean *A. femoralis* relative abundance and (A) silt content, (B) clay content, (C) basal area and (D) number of trees per module, taking detectability into account and excluding the outlier M11 (open circles; see the main text for more details). The relationships were statistically significant (percentage of silt: *r*^2^ = 0.69, *p* < 0.001; percentage of clay: *r*^2^ = 0.66, *p* < 0.001; basal area: *r*^2^ = 0.63, *p* < 0.001 and number of trees: *r*^2^ = 0.47, *p* < 0.01).

Simple linear regressions at the module level showed that the relative abundance of *A. femoralis* was negatively related to silt content (*F*_1.11_ = 27.28, *r*^2^ = 0.69, *p* < 0.001; Fig. 5A), basal area (*F*_1.11_ = 21.55, *r*^2^ = 0.63, *p* < 0.001; Fig. 5C) and number of trees (*F*_1.11_ = 11.77, *r*^2^ = 0.47, *p* < 0.01; Fig. 5D), and positively related to clay content (*F*_1.11_ = 24.78, *r*^2^ = 0.66, *p* < 0.001; Fig. 5B). Soil structure (silt and clay contents) explained up to 69% and 66%, and forest structure (basal area and number of trees) explained up to 63% and 47% of the variance in relative abundance of *A. femoralis*, respectively.

## Discussion

We show that soil characteristics and forest structure can predict the distribution and relative abundance of the widespread forest-dwelling Amazonian frog *A. femoralis* at a range of spatial scales. The species is generally patchily distributed, and its occurrence and relative abundance is associated with gradual variation in environmental features. At a large geographic scale, we revealed that *A. femoralis* is more abundant in open forests and in areas with clay-rich soils. Due to the lack of RAPELD modules in the savanna-open lowland forest transition area in the southwest of the PMI, this region remained unstudied. Dense forests are likely to inhibit dispersal, but the edges appear to provide suitable habitat for *A. femoralis* to reproduce, and these habitats may therefore act as corridors. Soil characteristics predict both forest structure (Cintra et al., 2013; Martins et al., 2014) and the presence of surface water (Woinarski, Fisher & Milne, 1999; Menin, Waldez & Lima, 2011) a requirement for reproduction by anurans with aquatic tadpoles such as *A. femoralis*. The physical composition of the soil is thus fundamentally interlinked with the occurrence of Amazonian frog species that depend on small stagnant water bodies on the forest floor.

Our estimated detection probabilities demonstrate that multiple surveys of *A. femoralis* in the PMI are highly unlikely to result in false negatives (recorded absences when the species is actually present). We expected this result because *A. femoralis* is easily detectable even when it is scarce, especially because males respond reliably to playback calls (Amézquita, Castellanos & Hödl, 2005; Amézquita et al., 2006; Betancourth-Cundar et al., 2016). Although the modules in the northeast (M1–M2 and M6–M9) show a more dispersed occupation of *A. femoralis* across plots, the average detectability did not markedly differ from modules of the southwest region (M10–M14) that have higher occurrences and relative abundances. In the Amazon basin, the minimum number of surveys necessary to determine the presence or absence of an amphibian species is not specified through general guidelines. As a consequence, for example environmental consultants regularly conduct only a single survey per locations, which can result in detection failure and therefore generate erroneous predictions of species’ responses to habitat factors (Gu & Swihart, 2003; Mazerolle et al., 2007). Our results suggest that the sampling regime applied for the present study (four surveys) is sufficient for the accurate detection of *A. femoralis*, although due to species-specific life histories this guide cannot be universally applied to other taxa (Smith et al., 2014).

The use of environmental proxies for estimating the occupancy of particular species has received some criticism, especially regarding the lack of consideration of behavioral interactions and the relevance of spatial scale (Stephens et al., 2015). The data used to evaluate species distributions in predictive models are often spatially biased and rarely include abundance (Warren, 2012), and the effects of biotic interactions are expected to be averaged out at broader scales (Fraterrigo, Wagner & Warren, 2004). Our data are not subject to these problems because sampling was carried out in a spatially stratified and consistent manner using the RAPELD research modules. Furthermore, data were obtained at large enough spatial scales in a hierarchical framework to test whether soil and forest characteristics measured in situ act as proxies for explaining the distribution and relative abundance of *A. femoralis* across a substantial part of its distribution. Due to the unavailability of module-specific precipitation data we are unable to include information on rainfall in our models, although they would be useful to predict whether soil ditches and other structures suitable for pond formation are actually filled with water.

Across the Amazon basin, differences in forest structure attributed to the physical characteristics of soil cause endogenous disturbances (Quesada et al., 2012; Cintra et al., 2013; Schietti et al., 2016), whereas edaphic or climatic factors can cause exogenous disturbances (Espírito-Santo et al., 2010, Cintra et al., 2013; Schietti et al., 2016). The two dominant forest phytophysiognomies in the PMI are spatially correlated with rainfall gradients (Sombroek, 2001), where forests in drier areas have lower stem densities and higher mass of individual trees compared to wetter forests, which have higher stem densities and lower individual tree mass (Cintra et al., 2013; Schietti et al., 2016). For *A. femoralis*, we show that these spatial differences in macro- and microhabitats shape its occurrence and relative abundance at both large and small scales, likely promoting its spatial differentiation. Parapatric segregation of populations associated with different forest formations has also recently been shown for an arboreal anuran occurring in the PMI (*Osteocephalus taurinus*, Ortiz, Lima & Werneck, 2018).

The two main vegetation types in the PMI are related to geomorphology and the establishment of the current Amazonian drainage system (Rossetti, Toledo & Góes, 2005; Hoorn et al., 2010; Latrubesse et al., 2010; Nogueira, Silveira & Guimarães, 2013). Tributaries in the dense northern forest drain into the Madeira River depression, while southern tributaries located within open forests drain into the Purus river depression (Fig. 1). According to Rossetti, Toledo & Góes (2005), seasonally flooded areas are linked to Holocene terrains, and the two major sedimentary units deposited 47,000 and 27,000 years ago are represented by lowland dense rainforest and lowland open rainforest. Drainage dynamics in each basin differ, and this coupled with different sedimentary loads and deposition age also influences vegetation establishment (Cohen et al., 2014). Old and well-drained soils are highly weathered (Emilio et al., 2013), whereas the soils in the northeast of the PMI are characterized by young and poorly drained sediments. Seasonally-flooded and poorly-structured soils provide a poorer substrate for root development and anchorage, resulting in frequent disturbances and high tree mortality rates, and consequently more dynamic, younger, and denser forests (Castilho et al., 2006; Feldpausch et al., 2011; Quesada et al., 2012; Cintra et al., 2013; Schietti et al., 2016). On the other hand, more structured soils with higher clay content are associated with older forests, where trees have higher individual mass and spacing between trees is larger (Castilho et al., 2006; Feldpausch et al., 2011; Emilio et al., 2013). Therefore, soil type can act as an environmental filter, selecting for different tree-growth strategies and partitioning the forest into patches of vegetation that are structurally distinct (Cintra et al., 2013; Emilio et al., 2013; Schietti et al., 2016).

Soil type also reflects other attributes of the environment relevant to anurans. A study conducted in central Amazonia found higher production of litter in plane areas with clay-rich soils (Luizão et al., 2004). Other studies found higher species richness and abundance of anurans at sites with clay-rich soil, presumably linked to higher surface water availability (Hadden & Westbrooke, 1996; Woinarski, Fisher & Milne, 1999; Menin et al., 2007). Rain can create ponds isolated from streams on soil rich in clay (Menin, Waldez & Lima, 2011). Consequently, such soil is directly related to the availability of small standing water bodies (Menin, Waldez & Lima, 2011), a prerequisite for the reproduction of *A. femoralis* (Kaefer et al., 2012; Ringler, Hödl & Ringler, 2015). Gascon (1995) described the general advantages of natural pools for frogs, and Ringler, Hödl & Ringler (2015) demonstrated the impact of simulated peccary presence on *A. femoralis* population size, where installing artificial pools almost doubled the density of frogs within two years. Because assessing small water bodies that may serve as tadpole deposition sites is difficult over large areas, using proxies to predict the distribution and abundance of *A. femoralis* in environments such as the Amazon basin can save time and money. Clay-rich soils are also associated with a higher variety and density of terrestrial arthropods (Franklin, Magnusson & Luizão, 2005; Aguiar, Gualberto & Franklin, 2006), which generally provide food for leaf litter frogs. However, the relationships between invertebrates and soil texture have not been investigated in the PMI, and additional data are necessary to establish a link between prey density and *A. femoralis* occurrence and relative abundance.

The influence of environmental heterogeneity on the distribution of frogs in the Amazonian lowlands generally depends on reproductive modes and breeding habitats (Zimmerman & Bierregaard, 1986; Menin et al., 2007; Menin, Waldez & Lima, 2011; Landeiro, Waldez & Menin, 2014; Ferrão et al., 2018). *A. femoralis* is likely to have ecological requirements in common with other forest frogs with similar life histories. For example, the density of *A. sumtuosus* was positively related to the number of isolated pools on a local scale (Jorge et al., 2016), and the occurrence and relative abundance of tree frogs (*Scinax*), which use similar water bodies for reproduction, is also positively affected by soil silt content (Ferrão et al., 2018).

## Conclusion

Frogs face a range of threats, including habitat loss and fragmentation, disease, and introduced species (Kats & Ferrer, 2003; Stuart et al., 2004; Lips et al., 2006; Becker et al., 2007). Given the pace of development in the Amazon Basin and the limited resources available for conservation, quick and cost-effective methods for predicting anthropogenic impacts are required. Our assessment of *A. femoralis* revealed its patchy distribution, with higher occupancy probabilities in areas with high, open vegetation and clay-rich soil. Identifying relatively easy-to-measure environmental features that reflect the distribution and abundance of suits of organisms, including species-specific detection probabilities, are of clear value to ecologists and conservation managers.

## Supplemental Information

10.7717/peerj.5424/supp-1 Supplemental Information 1 Supporting Information. Table S1. Locality and coordinates for each modules within the Purus–Madeira interfluvium.
Table S2. Sum of *A. femoralis* and soil and forest-structure properties in 90 plots located along the Purus-Madeira interfluve, in central-southern Amazonia.
Fig. S1. Diagram of the RAPELD model research modules, showing all distances: between each module, between the plots and between the two 5 km trails and the altitudinal contour lines. Source: Biodiversity Research Program (PPBio).

## Additional Information and Declarations

### Field Study Permissions

The following information was supplied relating to field study approvals (i.e., approving body and any reference numbers): The research project was approved by the appropriate governmental bodies: Ministério do Meio ambiente (MMA), through the Instituto Chico Mendes de Conservação da Biodiversidade (ICMBio license 13777) and Sistema de Autorização e Informação em Biodiversidade (SISBIO license 7836-1) for the sampling of A. femoralis.

### Data Availability

The following information was supplied regarding data availability: The raw data are provided in the Supplemental File.
