# Supplementary material for: Soil and forest structure predicts large-scale patterns of occurrence and local abundance of a widespread Amazonian frog
Source: PeerJ. 2018 Aug 9;6:e5424. doi: 10.7717/peerj.5424 (PMC6087616; doi:10.7717/peerj.5424)
Supplement: Supplemental Information 1 — Table S1. Locality and coordinates for each modules within the Purus–Madeira interfluvium. Table S2. Sum of A. femoralis and soil and forest-structure properties in 90 plots located along the Purus-Madeira interfluve, in central-southern Amazonia. Fig. S1. Diagram of the RAPELD model research modules, showing all distances: between each module, between the plots and between the two 5 km trails and the altitudinal contour lines. Source: Biodiversity Research Program (PPBio). [file peerj-06-5424-s001.docx]

## Supporting Information

**Soil and forest structure determine the large-scale occurrence and local abundance of a widespread Amazonian frog**

Anthony S. Ferreira, Robert Jehle, Adam J. Stow, and Albertina P. Lima

**Table S1.** Locality and coordinates for each modules within the Purus–Madeira interfluvium.

| **Modules** | **Municipality** | **Latitude** | **Longitude** |
| --- | --- | --- | --- |
| M01 | Careiro da Várzea | -3°37’S | -59°86’W |
| M02 | Careiro | -3°68’S | -60°34’W |
| M03 | Careiro | -4°13’S | -60°74’W |
| M04 | Borba | -4°38’S | -60°96’W |
| M05 | Beruri | -4°62’S | -61°24’W |
| M06 | Manicoré | -5°01’S | -61°55’W |
| M07 | Beruri | -5°28’S | -61°93’W |
| M08 | Manicoré | -5°63’S | -62°19’W |
| M09 | Tapauá | -5°96’S | -62°49’W |
| M10 | Humaitá | -6°57’S | -62°94’W |
| M11 | Humaitá | -7°21’S | -63°13’W |
| M12 | Jirau | -9°14’S | -64°52’W |
| M13 | Jirau | -9°16’S | -64°63’W |
| M14 | Abumã | -9°33’S | -64°74’W |

**
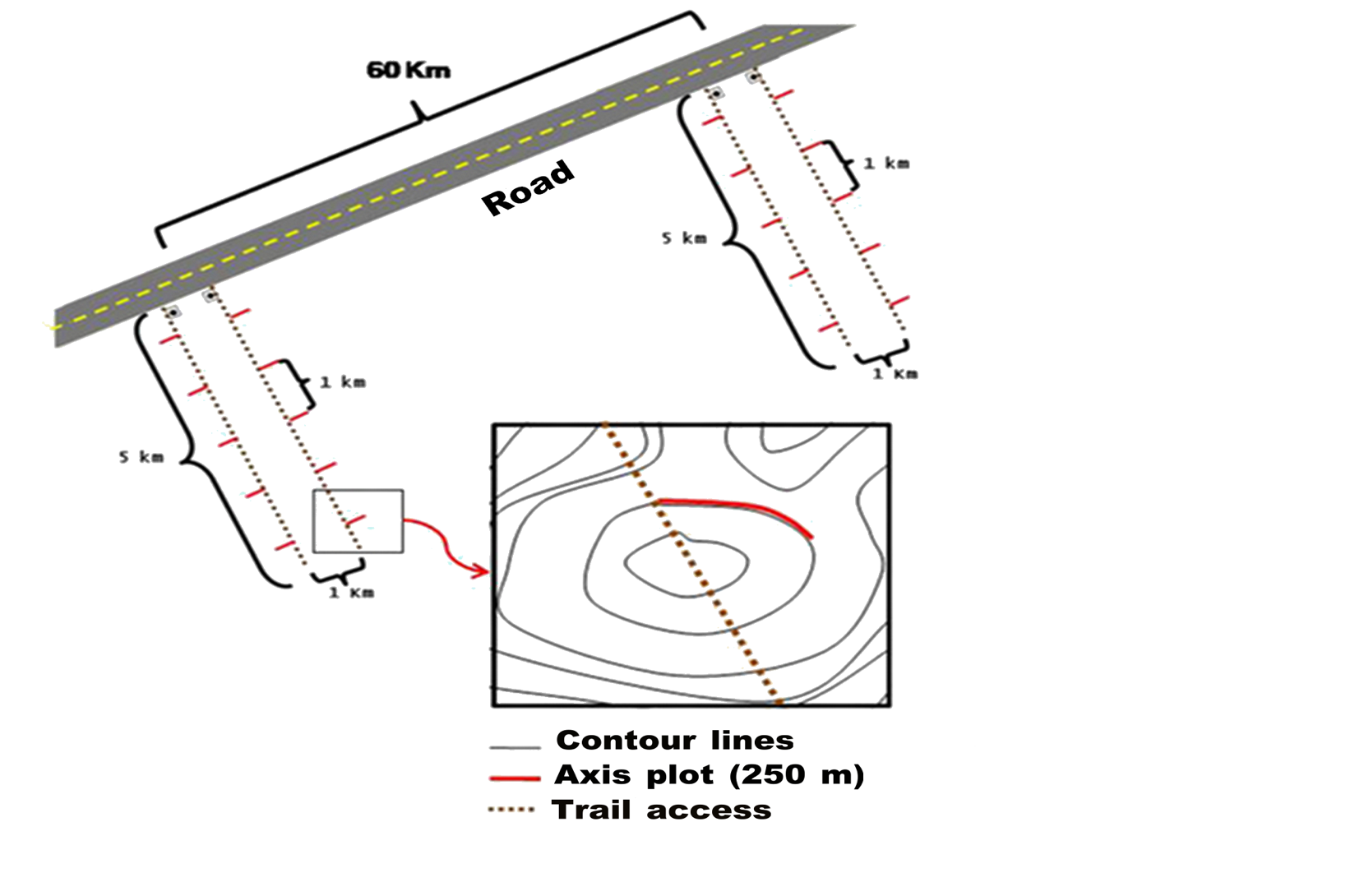
**

**Figure S1.** Diagram of the RAPELD model research modules, showing all distances: between each module, between the plots and between the two 5 km trails and the altitudinal contour lines. Source: Biodiversity Research Program (PPBio).

**Table S2.** Sum of recorded individuals of *A. femoralis* and soil and forest-structure properties in 90 plots located along the Purus-Madeira interfluve, in central-southern Amazonia.

| **Modules** | **Plots id** | **Sum of *A. femoralis*** | **Sand** | **Clay** | **Silt** | **Area basal** | **N° of trees** |
| --- | --- | --- | --- | --- | --- | --- | --- |
| M01 | TN_500 | 3 | 27.55 | 11.5 | 60.95 | 25.32 | 3861 |
| M01 | TN_1500 | 21 | 17.84 | 10.75 | 71.41 | 22.92 | 3562 |
| M01 | TN_2500 | 18 | 16.5 | 29.33 | 54.18 | 19.9 | 2051 |
| M01 | TN_4500 | 1 | 12.34 | 12.25 | 75.41 | 20.85 | 3573 |
| M02 | TN_500 | 1 | 20.53 | 18.5 | 60.98 | 28.13 | 4197 |
| M02 | TN_2500 | 0 | 21.65 | 13.25 | 65.1 | 25.41 | 3837 |
| M02 | TN_3500 | 0 | 20.68 | 21.25 | 58.08 | 26.41 | 5331 |
| M02 | TN_4500 | 12 | 15.36 | 16 | 68.64 | 33.12 | 5112 |
| M03 | TN_4500 | 0 | 12.83 | 28.5 | 58.67 | 31.97 | 7920 |
| M03 | TS_500 | 0 | 36.11 | 18.75 | 45.15 | 28.65 | 7621 |
| M03 | TS_2500 | 0 | 3.95 | 41.5 | 54.55 | 22.05 | 3505 |
| M03 | TS_3500 | 0 | 31.92 | 24.75 | 43.33 | 27.4 | 6773 |
| M04 | TN_500 | 0 | 12.31 | 26 | 61.69 | 28.6 | 7533 |
| M04 | TN_1500 | 0 | 25.55 | 16 | 58.45 | 33.37 | 9091 |
| M04 | TN_2500 | 0 | 12.39 | 12.5 | 75.11 | 32.4 | 7950 |
| M04 | TN_3500 | 0 | 30.23 | 13.75 | 56.03 | 35.1 | 8408 |
| M04 | TN_4500 | 0 | 43.04 | 6.25 | 50.71 | 33.84 | 9651 |
| M05 | TN_500 | 0 | 50.85 | 13 | 36.15 | 27.95 | 8478 |
| M05 | TN_1500 | 0 | 30.6 | 10.5 | 58.9 | 37.19 | 8257 |
| M05 | TN_2500 | 0 | 22.84 | 20 | 57.16 | 34.9 | 8507 |
| M05 | TN_3500 | 0 | 22.28 | 12.5 | 65.22 | 37.28 | 10506 |
| M05 | TN_4500 | 0 | 27.93 | 14 | 58.08 | 38.9 | 11475 |
| M06 | TN_500 | 0 | 35.22 | 8.25 | 56.54 | 34.82 | 6359 |
| M06 | TN_1500 | 0 | 43.2 | 17.75 | 39.06 | 34.53 | 8160 |
| M06 | TN_2500 | 3 | 32.89 | 19.38 | 47.74 | 29.71 | 6609 |
| M06 | TN_3500 | 5 | 29.58 | 27 | 43.43 | 35.86 | 7801 |
| M06 | TN_4500 | 0 | 19.38 | 15.5 | 65.13 | 35.22 | 8312 |
| M07 | TS_500 | 0 | 27.8 | 28.75 | 43.45 | 30.93 | 5788 |
| M07 | TS_1500 | 6 | 35.33 | 13.5 | 51.17 | 31.82 | 7227 |
| M07 | TS_2500 | 15 | 25.91 | 24.25 | 49.84 | 34.24 | 8240 |
| M07 | TS_3500 | 0 | 38.74 | 15.25 | 46.02 | 33.66 | 8052 |
| M07 | TS_4500 | 3 | 38.16 | 11.75 | 50.09 | 35.83 | 8379 |
| M08 | TS_500 | 1 | 36.88 | 19.25 | 43.87 | 33.46 | 5725 |
| M08 | TS_1500 | 0 | 28.74 | 14.25 | 57.02 | 27.17 | 7365 |
| M08 | TS_2500 | 6 | 13.86 | 19 | 67.14 | 31.58 | 6948 |
| M08 | TS_3500 | 1 | 39.72 | 10.75 | 49.54 | 29.45 | 5970 |
| M08 | TS_4500 | 0 | 17.62 | 13.75 | 68.63 | 33.51 | 6828 |
| M09 | TS_500 | 4 | 16.86 | 16 | 67.15 | 32.53 | 6945 |
| M09 | TS_1500 | 0 | 30.95 | 21.25 | 47.81 | 31.61 | 9149 |
| M09 | TS_2500 | 1 | 26.01 | 19 | 55 | 30.88 | 9609 |
| M09 | TS_3500 | 3 | 51.88 | 11.5 | 36.63 | 32.77 | 9997 |
| M09 | TS_4500 | 9 | 22.31 | 26 | 51.7 | 31.36 | 10096 |
| M10 | TS_500 | 1 | 47.42 | 13.5 | 39.08 | 32.3 | 6980 |
| M10 | TS_1500 | 7 | 40.89 | 12.5 | 46.61 | 32.53 | 4766 |
| M10 | TS_2500 | 3 | 35.95 | 14 | 50.06 | 27.8 | 5882 |
| M10 | TS_3500 | 10 | 42.92 | 11.75 | 45.33 | 28.44 | 4432 |
| M10 | TS_4500 | 7 | 42.32 | 22.75 | 34.93 | 30.63 | 5011 |
| M11 | TN_500 | 40 | 47.29 | 12.5 | 40.21 | 28.02 | 4676 |
| M11 | TN_1500 | 18 | 49.46 | 27.75 | 22.79 | 29.82 | 3330 |
| M11 | TN_2500 | 49 | 21.3 | 16.25 | 62.46 | 26.77 | 4180 |
| M11 | TN_3500 | 55 | 13.65 | 16.5 | 69.85 | 27.83 | 3631 |
| M11 | TN_4500 | 6 | 18.4 | 19.25 | 62.35 | 24.65 | 4090 |
| M12 | T1_0 | 8 | 14.4 | 77.7 | 7.9 | 17.46 | 1500 |
| M12 | T1_500 | 14 | 15 | 74.8 | 10.2 | 13.62 | 1349 |
| M12 | T1_1000 | 23 | 22 | 70.4 | 7.6 | 16.01 | 1591 |
| M12 | T1_2000 | 5 | 19.8 | 72.8 | 7.4 | 10.34 | 1813 |
| M12 | T1_3000 | 0 | 17.6 | 75.4 | 7 | 18.93 | 3215 |
| M12 | T1_5000 | 26 | 17.7 | 70.8 | 11.5 | 13.69 | 2612 |
| M12 | T2_0 | 33 | 19.3 | 69 | 11.7 | 11.88 | 1335 |
| M12 | T2_500 | 19 | 18.5 | 62.2 | 19.3 | 11.42 | 1902 |
| M12 | T2_1000 | 16 | 18.8 | 70.4 | 10.8 | 14.04 | 2294 |
| M12 | T2_2000 | 7 | 16.6 | 70.4 | 13 | 10.97 | 2425 |
| M12 | T2_3000 | 0 | 19.3 | 64.1 | 16.6 | 14.06 | 2629 |
| M12 | T2_4000 | 11 | 19.9 | 62.6 | 17.5 | 27.33 | 2264 |
| M12 | T2_5000 | 10 | 17 | 64.2 | 18.8 | 24.62 | 1989 |
| M13 | T1_500 | 40 | 19.5 | 72.1 | 8.4 | 26.87 | 8011 |
| M13 | T1_1000 | 24 | 20.5 | 70.5 | 9 | 19.67 | 3410 |
| M13 | T1_2000 | 13 | 22 | 71 | 7 | 20.41 | 3288 |
| M13 | T1_3000 | 11 | 23.4 | 69.1 | 7.5 | 26.72 | 3618 |
| M13 | T1_4000 | 32 | 20.8 | 71.2 | 8 | 20.75 | 3529 |
| M13 | T1_5000 | 5 | 18.7 | 72.3 | 9 | 22.06 | 5155 |
| M13 | T2_1000 | 50 | 32 | 59.2 | 8.8 | 22.33 | 3373 |
| M13 | T2_2000 | 0 | 31.1 | 59.8 | 9.1 | 32.83 | 2665 |
| M13 | T2_3000 | 1 | 28 | 64.2 | 7.8 | 12.44 | 3497 |
| M13 | T2_4000 | 7 | 31.7 | 58.7 | 9.6 | 11.91 | 4031 |
| M13 | T2_5000 | 4 | 27.5 | 64.7 | 7.8 | 9.73 | 4023 |
| M14 | T1_0 | 6 | 30.8 | 52.6 | 16.6 | 20.47 | 2521 |
| M14 | T1_500 | 0 | 22.5 | 61.7 | 15.8 | 20.98 | 2176 |
| M14 | T1_1000 | 1 | 27 | 54.5 | 18.5 | 16.55 | 3466 |
| M14 | T1_2000 | 0 | 19.8 | 63.2 | 17 | 17.9 | 5742 |
| M14 | T1_3000 | 0 | 17.5 | 69.5 | 13 | 17.67 | 3391 |
| M14 | T1_4000 | 2 | 20.4 | 66.8 | 12.8 | 15.79 | 2731 |
| M14 | T1_5000 | 0 | 29.1 | 53.9 | 17 | 18.7 | 3467 |
| M14 | T2_0 | 48 | 26.6 | 60.4 | 13 | 16.22 | 4971 |
| M14 | T2_500 | 56 | 31.8 | 49.2 | 19 | 17.63 | 3232 |
| M14 | T2_1000 | 20 | 27.4 | 58.1 | 14.5 | 18.4 | 4218 |
| M14 | T2_2000 | 0 | 30.5 | 52.3 | 17.2 | 13.5 | 7769 |
| M14 | T2_3000 | 0 | 32 | 49.2 | 18.8 | 14.56 | 5829 |
| M14 | T2_4000 | 14 | 31.7 | 52.3 | 16 | 16.03 | 6582 |
| M14 | T2_5000 | 0 | 40 | 48 | 12 | 18.14 | 6004 |
|  | | | | | | | |
